# Physiotherapy for people with painful peripheral neuropathies: a narrative review of its efficacy and safety

**DOI:** 10.1097/PR9.0000000000000834

**Published:** 2020-09-23

**Authors:** Tom Jesson, Nils Runge, Annina B. Schmid

**Affiliations:** a Division of Surgery and Interventional Science, University College London, London, United Kingdom; b Connect Health, Benton Lane, Newcastle upon Tyne, Tyne and Wear, United Kingdom; c Nuffield Department for Clinical Neurosciences, University of Oxford, Oxford, United Kingdom

**Keywords:** Physiotherapy, Physical therapy modalities, Physical therapy, Exercise, Manual therapy, Neuropathic pain, Neuropathy, Radicular pain, Chemotherapy-induced peripheral neuropathy

## Abstract

Pharmacological treatment for peripheral neuropathic pain has only modest effects and is often limited by serious adverse responses. Alternative treatment approaches including physiotherapy management have thus gained interest in the management of people with peripheral neuropathies. This narrative review summarises the current literature on the efficacy and safety of physiotherapy to reduce pain and disability in people with radicular pain and chemotherapy-induced peripheral neuropathy, 2 common peripheral neuropathies. For chemotherapy-induced peripheral neuropathy, the current evidence based on 8 randomised controlled trials suggests that exercise may reduce symptoms in patients with established neuropathy, but there is a lack of evidence for its preventative effect in patients who do not yet have symptoms. For radicular pain, most of the 21 trials investigated interventions targeted at improving motor control or reducing neural mechanosensitivity. The results were equivocal, with some indication that neural tissue management may show some benefits in reducing pain. Adverse events to physiotherapy seemed rare; however, these were not consistently reported across all studies. Although it is encouraging to see that the evidence base for physiotherapy in the treatment of peripheral neuropathic pain is growing steadily, the mixed quality of available studies currently prevents firm treatment recommendations. Based on promising preliminary data, suggestions are made on potential directions to move the field forward.

## Abstract 2

Supplemental Digital Content is Available in the Text.

The efficacy and safety of physiotherapy in the management of people with peripheral neuropathies is explored. Potential ways forward based on promising data are suggested.

## 1. Introduction

Peripheral neuropathic pain is common with an estimated population prevalence of 6.9% to 10%.^34^ Neuropathic pain is often associated with peripheral neuropathies, which can be systemic or focal in nature. Common systemic neuropathies include chemotherapy-induced neuropathy and diabetic neuropathy. The most prevalent focal neuropathies include entrapment neuropathies such as carpal tunnel syndrome, radiculopathy, and radicular pain. To date, treatment for patients with peripheral neuropathies and neuropathic pain has relied largely on pharmacology. However, recent reviews suggest that the efficacy of neuropathic pain medications is only modest and often accompanied by adverse drug responses.^23,39^ Physiotherapy management for patients with peripheral neuropathies has gained increasing interest in the past decade, but evidence for its efficacy and safety is only starting to emerge.^21,42,74^ The aim of this narrative review is to describe the current evidence for the efficacy and safety of physiotherapy to reduce pain and disability in patients with peripheral neuropathies and to suggest future directions for research. We focused on one systemic and one focal peripheral neuropathy: chemotherapy-induced peripheral neuropathy (CIPN) and radicular pain. Although this is a narrative review, we used a thorough approach including risk of bias assessment to identify the relevant literature and assess its methodological quality (Appendix 1, available at http://links.lww.com/PR9/A70; supplementary Table S1 and S2, available at http://links.lww.com/PR9/A68 and http://links.lww.com/PR9/A69).

## 2. Physiotherapy for chemotherapy-induced peripheral neuropathy

The underlying pathophysiology of CIPN is complex, not fully understood, and varies depending on the causative agent.^10^ Key mechanisms include microtubule disruption, mitotoxicity, and the neuroimmune response, usually affecting sensory nerves as a length-dependent “glove and stocking” polyneuropathy.^10^ Chemotherapy-induced peripheral neuropathy is a major concern in the treatment of cancer because it is a dose-limiting factor for chemotherapy and has a severe and persistent impact on quality of life (QoL).^41^

People with CIPN might experience pain or a combination of numbness, tingling, or hot or cold sensations better described as “unpleasant” symptoms. Reflecting this, some of the available studies on physiotherapy in CIPN used broader measures of unpleasant sensory symptoms instead of specific pain measurement tools. In these cases, we concentrated on the most relevant subscore of these tools. Throughout this review, we will often refer to “CIPN pain” as an umbrella term that includes these unpleasant symptoms.

Table 1 summarises studies on physiotherapy for patients with CIPN. These can be broadly divided into 2 categories: those aimed to attenuate the development of CIPN during chemotherapy, and those aimed to reduce the symptoms of already-established CIPN.

**Table 1:** Details of included studies for chemotherapy-induced peripheral neuropathy.

	Participants	N (at follow-up)	Intervention	Control	Pain primary outcome?	QoL outcome	Results (pain)	Risk of bias
Streckmann et al.^73^	People with lymphoma scheduled for chemotherapy	61 (51)	36-wk, twice-weekly supervised aerobic, sensorimotor and strength training, in addition to usual care including physiotherapy (not detailed)	Usual care including physiotherapy	Yes, as a subset of QoL measure	The authors report statistically significant changes on the EORTC-QoL-C30 but do not provide the values of these changes or between-group differences	After 36 wk there was an 8-point mean difference between groups on the pain subsection of the 100point EORTC-QoL scale (*P* = 0.396). Baseline values not provided.	High
Kleckner et al.^44^	People with different kinds of cancer, mostly breast cancer, undergoing chemotherapy.	456 (355)	Six-week, daily, mixed progressive exercise programme according to the American College of Sports Medicine guidelines for exercise for cancer patients.	Usual care	No	Not reported	After 6 wk, there was a mean difference of −0.42 in numbness and tingling on a 10-point scale (95% CI: −0.85 to 0.02) in favour of the exercise group, and a mean difference of −0.46 (95% CI: −0.01 to −0.91) for hot/cold sensations in the hands and feet.	High
Vollmers et al.^75^	People with breast cancer undergoing chemotherapy	43 (36)	Sensorimotor and strength exercise programme performed twice per week during chemotherapy and continued for 6 wk afterwards	Usual care	No	The authors reported “hardly any significant difference” on the EORTC-QoL-C30, but did not provide any values.	“No difference” reported. No further data provided.	High
Zimmer et al.^81^	People with metastasised colorectal cancer and CIPN, undergoing further chemotherapy	30 (24)	Eight-week, twice-weekly, supervised aerobic, balance and resistance exercise programme	Usual care	Yes, as part of neurotoxicity construct	On the 100-point Trial Outcome Index of the FACT/GOG scale, the intervention group scores remained stable during chemotherapy whereas the control group scores worsened by 7.14 points (*P* = 0.077) at the end of treatment and 8.07 points (*P* = 0.037) at 4-wk follow-up.	After the intervention, symptoms of neurotoxicity had worsened during chemotherapy by 5.1 points on the 44-point FACT/GOG scale (*P* = 0.045), whereas for patients in the exercise group, symptoms improved by 2.12 points, (*P* = 0.023); between-group difference *P* = 0.002.	Some concerns
Bland et al.^8^ (2018)	Women with breast cancer undergoing taxane chemotherapy	31 (25)	One group engaged in supervised general exercise 3 d per week for the duration of chemotherapy. The other group engaged in the same programme after cessation of chemotherapy.	None (2 intervention arms)	Yes, as a subset of QoL measure	At the end of chemotherapy treatment, the early exercise group reported a mean 11.9 points higher QoL (*P* 0.05) on the EORTC QLQ-C30. 10–15 wk after chemotherapy, when the second group had also exercised, there were no significant differences.	“No [significant] difference” reported. Data are provided in graph form but no numerical data are provided on the mean difference between groups for sensory symptoms.	High
Dhawan et al.^16^	People with cancer (not specified) and CIPN undergoing paclitaxel and carboplatin chemotherapy.	45 (41)	10-wk daily mixed exercise programme, performed unsupervised at home.	Usual care	Yes	The authors present data for symptom-related QoL, functional QoL, and global health QoL. After 10 wk, for all measures, there is a greater than 10-point mean difference in favour of the exercise group. All differences have *P* < 0.05	After the intervention, patients in the intervention group reported 83.1 points on the 279 point CIPNAT scale of CIPN symptom experience, compared to 140.8 points in the control group. Between-group difference *P* < 0.0001. On the LANSS neuropathic pain scale of 0–24, the intervention group reported 10.7 and the CG 15.8. Between-group difference *P* = 0.001.	Some concerns
Izgu et al.^38^	Women with breast cancer undergoing paclitaxel chemotherapy.	40 (40)	30 min of Swedish massage once per week before chemotherapy, for 12 wk.	Usual care	Yes	The authors report 3 subdimensions of the EORTC QLQ CIPN20 with 3 subdimensions—autonomic, sensory, and motor. The intervention group means are greater than the control group means by >10 points at 12 wk only. The authors do not provide precise between-group differences.	Throughout treatment, the proportion of patients reporting neuropathic pain on the S-LANSS scale remained at the baseline level of 10.5% in the treatment group, but increased in the control group. The greatest difference between the groups was at 12 wk, when 57% of the control group reported neuropathic pain (*P* = 0.006); by 16 wk this dropped to 38% (*P* = 0.069).	Some concerns
Kneis et al.^45^	People with CIPN with different kinds of cancer, mostly breast cancer and colorectal cancer, who had finished chemotherapy.	50 (41)	Twelve weeks of twice-weekly supervised balance and endurance training	Endurance training only	Yes	The authors report no significant difference between groups on the EORTC QLQ-C30. They do not give exact values.	The authors present a per-protocol analysis of 37 patients. Both groups improved by a median difference of 7 points on the 100 point sensory subscale of the EORTC-QLQ (95% CI −15 to 0). On the 44 point neurotoxicity subscale of the FACT&GOG, the intervention group worsened by a median value of 3 points (95% CI 1–6) and the control group by 2 points (95% CI 0–4)	Some concerns

The number of participants shown at follow-up is for the longest-term follow-up in each trial.

95% CI, 95% confidence interval; CIPN, chemotherapy-induced peripheral neuropathy; CIPNAT, Chemotherapy-Induced Peripheral Neuropathy Assessment Tool; EORTC-QLQ, European Organisation for Research and Treatment of Cancer Quality of Life Questionnaire; FACT/GOG, Functional Assessment of Cancer Therapy/Gynecologic Oncology Group; QoL, quality of life; S-LANSS, Self-Administered Leeds Assessment of Neuropathic Symptoms and Signs.

### 2.1. Physiotherapy in the prevention of chemotherapy-induced peripheral neuropathy related pain

Most prevention studies do not report a more meaningful pain reduction with physiotherapy interventions compared to a control intervention or standard care. For instance, Streckmann et al.^73^ tested the efficacy of a twice-weekly, 36-week aerobic, sensorimotor and strength training programme against usual care (which was not defined) for people with malignant lymphoma scheduled for chemotherapy. The results did not suggest a difference in pain between the 2 groups. Of note, recruitment for this trial fell short of the target of 240 patients with only 61 enrolled, so the risk of a false-negative finding is high. In a small proof-of-concept trial (n = 31), Bland et al.^8^ examined the effects of a mixed aerobic, resistance and balance exercise programme 3 times per week in a small sample of people with breast cancer. One group performed the exercise programme during their course of chemotherapy, the other group after. In the group who exercised during their chemotherapy, fewer patients reported numbness in their toes and feet for the first 3 weeks, but no clinically meaningful difference was observed between the groups for any other variable at any other time. Vollmers et al.^75^ (n = 36) tested a twice-weekly sensorimotor and strengthening program, started during chemotherapy and continued for 6 weeks after, against usual care. The authors reported “hardly any significant improvements” between groups on their sensory measures but did not report their data, focusing instead on postural sway and strength.

Two primary prevention studies did find evidence that physiotherapeutic treatment can attenuate the progression of CIPN symptoms, although these differences were small. Kleckner et al.^44^ performed a secondary analysis of a well-powered randomized controlled trial (RCT, n = 456) designed to assess the effects of adding a six-week walking and resistance exercise programme to standard medical care compared to standard medical care alone for people with breast cancer receiving chemotherapy. The nature of the standard medical care was not detailed in the study. The trial was designed to assess patients' fatigue, but the authors performed a secondary analysis of patients' CIPN symptoms in the 355 patients who completed the study. Both groups reported worsening hot and cold sensations and worsening numbness and tingling during the course of chemotherapy. At the end of the trial, the intervention group reported slightly less symptoms, but there was no evidence of a clinically meaningful difference between the 2 groups. Finally, Izgu et al.^38^ examined the effects of 30 minutes of Swedish massage before each session of chemotherapy added to usual care, compared to usual care alone in 40 patients with breast cancer. Throughout treatment, the proportion of patients reporting neuropathic pain on the Leeds Assessment of Neuropathic Symptoms and Signs (LANSS) scale remained at the baseline level of 10.5% in the treatment group but increased in the control group. The greatest difference between the groups was at 12 weeks, when 57% of the control group reported neuropathic pain but by 16 weeks, this dropped to 38%.

In summary, there are currently no data to suggest that physiotherapeutic treatments can prevent the development of CIPN symptoms to a clinically meaningful degree in patients who do not yet have symptoms. Of note, we consider this to be an absence of evidence, not evidence of absence. Specifically, most available trials had a high risk of bias and were either underpowered and/or did not measure pain or unpleasant symptoms as a primary endpoint. This increases the risk of accepting false-negative results (type II error). This problem is not unique to trials for physiotherapy for CIPN, but has also been documented in the literature on its pharmacological treatment.^27^

Besides trial design and quality, a number of other factors might explain the lack of observed effect. For one, physiotherapy interventions might not be effective in reducing patients' CIPN pain. Or, physiotherapy might work but fails to provide prolonged effects over many weeks of chemotherapy. Three studies observed an initial benefit for exercise that was lost by the end of the trial.^9,38,67^ A dilution effect is also likely to be a factor because a substantial proportion of patients do not develop CIPN during chemotherapy, thus reducing power to detect effects in these studies. Indeed, in the trial by Bland et al.,^8^ only half of the patients report symptoms and in the trial by Kleckner et al.,^44^ the average symptom rating at the end of the trial was less than 2 on a 10-point scale, potentially suggesting a dilution effect by those patients who did not develop neuropathic pain.

### 2.2. Physiotherapy for the treatment of established chemotherapy-induced peripheral neuropathy related pain

The few studies investigating physiotherapy treatment of already-established CIPN suggest that exercise might ease symptoms. Three studies examined the effect of exercise for patients with established CIPN, of which 2 compared exercise to usual care^16,81^ and one compared 2 different types of exercises.^45^ First, Zimmer et al.^81^ (n = 30) tested an 8-week, twice-weekly mixed exercise programme, consisting of balance, coordination, endurance, and resistance training against usual care in participants with CIPN who were undergoing chemotherapy for metastasised colorectal cancer. For patients in the usual care group, symptoms of neurotoxicity worsened during chemotherapy by 5.1 points on a 44-point scale, whereas for patients in the exercise group, symptoms improved by 2.12 points, meeting a minimal clinically important difference for this scale.^78^ Second, Dhawan et al.^16^ (n = 45) tested the effects of a ten-week strength and balance home exercise program compared to usual care in patients undergoing chemotherapy. Compared to the usual care group, the intervention group reported lower neuropathic pain scores (LANSS scale) and less symptoms as measured by the CIPN Assessment Tool scale. This was despite the intervention group receiving a higher chemotherapeutic dose than the usual care group. Third, Kneis et al.^45^ tested a twice-weekly exercise bike and balance programme against an exercise bike programme alone in a total of 50 patients. They did not find evidence of a difference between groups in scores on symptom- or neurotoxicity-related tools. Although both groups improved from baseline, the absence of a no-treatment group leaves it unclear as to whether this may be attributed to the interventions or reflects natural history.

In summary, the few randomised controlled trials available suggest that exercise might ease symptoms for patients who already have CIPN. This is supported by a prospective cohort study with a “control” period followed by 8 weeks of exercise intervention in patients with established CIPN.^56^ In this study, the exercise intervention improved symptoms, balance, mobility, and QoL compared to the control period. Because the available studies were small and none had a low risk of bias, it is not possible to draw a firm conclusion. Nevertheless, physiotherapy for people with established CIPN seems to be a promising direction for future research.

### 2.3. Effects of physiotherapy on quality of life in chemotherapy-induced peripheral neuropathy

Our intention with this narrative review was to report data on disability, but most trials examining CIPN measured QoL rather than disability so we have focused on this instead. The data on the effects of physiotherapy on QoL follow a similar pattern to those on pain. With one exception, there is a lack of data on the effects of physiotherapy to prevent deterioration in QoL in people at risk of developing CIPN. Three studies in this category did not report QoL outcomes in enough detail to determine whether there was a meaningful difference between groups.^44,73,75^ Izgu et al.^38^ reported that massage improved QoL when added to usual care, but this improvement decreased after 12 weeks. Only Bland et al.^8^ reported a clinically meaningful,^78^ sustained, albeit small improvement in QoL for patients who exercised during chemotherapy. For the effects of physiotherapy on QoL for people with established CIPN, the data suggest a promising effect. Zimmer et al.^81^ and Dhawan et al.^16^ both reported a meaningful benefit, whereas Kneis et al.^45^ did not find any difference between groups.

### 2.4. Safety of physiotherapy for chemotherapy-induced peripheral neuropathy

It seems that physiotherapy is generally safe in the treatment of CIPN with only one minor, transient adverse event reported across all trials: haematuria in an exercising patient with a urethral stent.^81^ Although all studies reported on adverse events, only 2 studies^44,75^ defined an adverse event or explained how these data were collected, highlighting the importance of careful reporting in future trials.

### 2.5. Summary and future direction for physiotherapy in chemotherapy-induced peripheral neuropathy

There is a lack of evidence that physiotherapeutic treatments can attenuate the development of CIPN in people who do not yet have symptoms, but there is some evidence that exercise could be beneficial for patients with already established CIPN. None of the studies we discussed here had a low risk of bias (supplementary Table S1, available at http://links.lww.com/PR9/A68). Albeit preliminary, these findings may help clinicians direct treatment until firmer evidence is available.

A recent systematic review^19^ drew a stronger conclusion that exercise is likely to be effective for symptoms of CIPN. This review was not limited to RCTs, but included 2 single-arm trials that did not meet our inclusion criteria.^20,76^ Another recent integrative review concluded that exercise is “safe, feasible, and potentially effective for patients with CIPN” but that a definitive conclusion could not be drawn from the evidence available.^42^

The preliminary promising results from some trials warrant further well-powered studies to conclusively examine the role of physiotherapy in the prevention or treatment of CIPN. At the recent National Cancer Institute Clinical Trials Planning Meeting,^18^ exercise was described as 1 of the 3 most promising interventions for CIPN. On one clinical trial registry (clinicaltrials.gov), we found 4 ongoing clinical trials testing the efficacy of sensorimotor training and strength (NCT02871284), therapeutic ultrasound (NCT02499939), physiotherapy care (NCT02239601), and aerobic walking (NCT03515356) in patients with CIPN. These studies will hopefully clarify the role physiotherapy may play in the management and prevention of CIPN.

## 3. Physiotherapy for radicular pain

Radicular pain is caused by mechanical (typically compressive) or chemical irritation of a spinal nerve or nerve root, most commonly in the lumbar or cervical spine. The natural history of radicular pain is often favourable, but it can become chronic in about a third of patients.^33,37,46^ Typical causes of radicular pain are disk herniations, spinal stenosis, and spondylolisthesis. Classically, patients will report pain in the affected dermatome, although in practice, pain can be isolated to patches of the lower quadrant and extradermatomal pain is the rule rather than the exception.^60^ The term “radicular pain” refers to the painful aspect of nerve compromise, whereas the term “radiculopathy” refers to the loss of sensory and motor function that can sometimes be observed.^9^ In addition, some patients have radiating axial/limb pain as a result of neural mechanosensitivity, which can be nociceptive in nature.^70^ These distinctions are often not made in trials examining treatments for radiating leg pain.^51^ Because our review focuses on pain, we included studies that reported on patients with radicular pain or neural mechanosensitivity with and without radiculopathy.

To guide the readers through the evidence, we grouped the studies on physiotherapy for radicular pain into 3 categories: those that investigate interventions that aim to stabilise or improve motor control of the spine, those that investigate exercises or manual therapy aimed at reducing neural mechanosensitivity, and a remaining category that investigates interventions that are too heterogeneous to classify. Within each category, we also considered whether studies compared each intervention to a control group of minimal care, or to a more substantial control intervention. The details of studies for physiotherapy for radicular pain are summarised in Table 2.

**Table 2:** Details of included studies for radicular pain.

	Participants	N (at follow-up)	Intervention	Control	Disability	Results (pain)	Risk of bias
Persson et al.^63^	People with chronic cervical radiculopathy with evidence of nerve compression on MRI	82 (79)	3 mo of twice per week physiotherapy including TENS, heat, cold, massage, active and aerobic exercise chosen at the discretion of the 25 physiotherapists involved. Another intervention arm received decompression surgery	Comfortable rigid collar, worn for 3 mo	Not measured	Fourteen weeks after treatment began, mean pain intensity in the surgery arm had improved by −20 points on a 0–100 VAS. Improvement was −9 points in the physiotherapy group, and −1 point in the neck collar group. Between-group differences were *P* < 0.01.	Some concerns
Hofstee et al.^35^	People with acute lumbar radicular pain	250 (225)	Four to 8 wk of twice per week physiotherapy including manual mobilisations and spinal exercise. Another intervention group received advice to rest in bed for 7 d, and to rest as much as possible thereafter	Advice to continue with normal activities of daily living	After 2 mo, the difference between the bed rest and the control group was a mean −2.7 (−9.9 to 4.4) on the 0–100 Quebec Disability Scale in favour of the control. There was no mean difference between the physiotherapy and the control group. 2 m: −0.0 (−7.2 to 7.3).	After 2 mo, the difference between the physiotherapy group and the control group was a mean 0.8 points on a 0–100 VAS (95% CI: −8.2 to 9.8). The difference between the bed rest and control arms was a mean 2.5 (95% CI: −6.4 to 11.4) in favour of the control group.	High
Bakhtiary et al.^4^	People more than 2 mo of radicular pain from a herniated lumbar disk, referred from orthopaedic care to physiotherapy	60 (52)	One group performed stabilisation exercises at home twice per day for 4 wk, whereas the other group did not exercise. Groups then crossed over.	None (2 intervention arms)	Not measured	After the first 4 wk, the exercising group improved by a mean difference from baseline of −3.2 points on a 10-cm VAS, whereas the group not exercising improved by −0.5 points; the between-group difference was −2.7 points (95% CI: −3.5 to −1.9). After 8 weeks, when the second group had also exercised, the between-group difference was −0.9 (95% CI: −1.7 to −0.01).	Some concerns
Luijsterburg et al.^53^	People with acute lumbar radicular pain in primary care	135 (117)	GP and physiotherapist care. Physiotherapy based on exercise and return to activity, with no manual therapy or electrotherapy.	Usual guideline-based GP care	After 12 wk, the difference between groups on the 24 point RMDQ was 0.8 (95% CI: −1.6 to 3.2) in favour of the control group. After 1 y, the difference was −0.9 (95% CI: −3.0 to 1.3) in favour of the intervention group.	After 12 wk, the intervention group reported an improvement in leg pain of −3.9 on a 10-point NRS and the control group reported an improvement of −3.7; the mean difference between the 2 groups of 0.3 (95% CI: −0.06 to 1.2). After 1 y, the intervention group reported an improvement of −4.4 and the control group −3.7; the mean difference between the 2 groups was −0.7 (95% CI: −1.7 to 0.2). The primary outcome measure was global perceived effect.	Low
Kuijper et al.^48^	People with acute cervical radicular pain	205 (192)	One intervention group wore a semihard collar during the day for 3 wk and were advised to rest as much as possible. They were then weaned from the collar for 3 wk. The other intervention group engaged in twice-weekly supervised physiotherapy for 6 wk, with a focus on mobilising and stabilising the spine, along with home exercises.	Reassurance.	After 6 weeks, the beta-coefficient for weekly change in the NDI was 0.8 points per week (95% CI: −1.8 to 0.2) in the physiotherapy group when compared to the control group.	The authors used generalised estimating equations to show that both intervention groups reported a benefit of 1.9 mm on a 100-mm VAS per week in arm pain for the first 6 wk (95% CIs −3.3 to −0.5 for neck collar; −3.3 to −0.8 for exercise). After 6 mo, all groups reported a median of 0 points.	High
Young et al.^79^	People with mixed acute and chronic cervical radicular pain	81 (69)	Manual therapy, exercise, and intermittent cervical traction	Manual therapy, exercise, and sham traction	After 4 weeks, there was a mean difference of 1.5 points (95% CI: −6.8 to 3.8) on the neck disability index and 0.29 points on the PSFS 0.29 (95% CI: −1.8 to 1.2) in favour of the intervention group.	After 4 weeks, there was an adjusted mean between-group difference of 0.52 points on a 10-point NRS (95% CI −1.8 to 1.2) in favour of the intervention group	Some concerns
Huber et al.^36^	People with acute lumbar radicular pain, caused by a herniated disk, in primary care	52 (52)	Three sessions per day for 20 d of supervised supine isometric exercises. Not specified what proportion of the sessions was supervised.	Advice to continue with activities of daily living	Not measured	After the intervention, the intervention group reported 1.7 points less pain on a 10-point VAS but the authors did not report the between-group difference or provide exact *P*-values for the within- or between-group differences, so we are unable to say how precise this estimate is.	High
Albert and Manniche^2^	People with mixed acute and chronic lumbar radicular pain, in secondary care	181 (170)	Information, advice, and symptom-guided spinal exercises based on McKenzie method of directional preference, along with stabilizing exercises. Eight weeks with 4–8 treatment sessions.	Information, advice, and low-dose general exercises to stimulate circulation	The authors reported no significant between-group differences on the RMDQ but did not give values for this.	After treatment, there was a 0.8-point mean difference between the groups on a 10-point NRS for patients' current leg pain (*P* = 0.06), in favour of the intervention group. On a “total leg pain” score, including current leg pain, worst leg pain, and average leg pain, there was no difference in mean ratings between the 2 groups (*P* value not provided).	High
Nee et al.^61^	People with more than 4 wk of nerve-related arm pain. With a positive median nerve tension test and without more than 2 abnormal neurological findings. Recruited from the community through advertisements in newspapers and e-newsletters	60 (56)	A standardised programme of 4 sessions of neural tissue management, including exercise, manual therapy, and education, over 4 wk.	Advice to continue with activities of daily living, with complementary treatment after the trial	After treatment, there was a mean 3.4-point difference between groups (95% CI: −0.6 to 6.3) on the NDI and a 2.1-point difference (95% CI: 0.9–3.2) on the PSFS, both in favour of the intervention group.	After treatment, there was a mean 1.5-point difference on a 0–10 NRS favouring the intervention group (95% CI: −0.5 to −2.6). Primary outcome measure was Global Rating of Change.	Low
Fritz et al.^26^	People with mixed acute and chronic cervical radicular pain	86 (54)	One intervention group performed exercise (scapula and neck muscle strengthening) and received mechanical traction. The other intervention group performed exercise and overdoor traction. 10 sessions over 4 wk.	10 sessions over 4 wk of exercise only	Immediately after treatment, there were small, not clinically or statistically significant differences between groups on the NDI. At 6-mo follow-up, the group receiving mechanical traction reported a mean of 13.3 points (95% CI: 5.6–21.0) less disability. The group performing overdoor traction reported a mean 5.2 points (95% CI: −2.6 to 13.0) less disability.	Immediately after treatment, there were small, not clinically or statistically significant differences between groups. At 6-mo follow-up, the group receiving mechanical traction reported a mean of 2.3 points (95% CI: 0.9–3.8) less arm pain on a 10-point NRS scale than the exercise-only control group. The group performing overdoor traction reported 2.5 points' less pain (95% CI: 1.0–4.0).	Some concerns
Langevin et al.^49^	People with cervical radicular pain	36 (36)	A 4-wk programme of manual therapy, exercises and stretches aimed at increasing space in the intervertebral foramen.	A similar programme not aimed at increasing space in the intervertebral foramen	After 4 wk there was a mean 2.3-point difference (95% CI: 10.1 to −5.5) between groups on the NDI and 3.9 points (95% CI: 14.0 to −6.2) on the QuickDASH, both in favour of the intervention group. After 8 wk, this was 4.6 points (12.1, −2.8) and 5.6 points (95% CI: 20.0 to −8.9), respectively.	After 4 wk there was a mean −0.1 difference (95% CI: −1.9 to 1.8) in mean arm pain on a 10-point NRS. After 8 wk, the difference between the groups was −1.3 points (95% CI: −2.8 to 0.2).	Some concerns
Moustafa and Diab^59^	People with chronic lumbar radicular pain from a disk herniation and anterior head translation as measured by cervical radiograph	154 (131)	A 2-y programme of gym-based “functional restoration” exercises in phases of decreasing independence and increased supervision. Some exercises were intended to encourage upright neck posture.	The same exercise programme without the exercises aimed at neck posture	After 10 weeks, the control group reported a mean 2.82 points lower score on the ODI (*P* = 0.08). After 2 y, the mean difference was 11.8 (*P* = 0.005).	After 10 weeks, the control group reported 0.2 points less leg pain on a 10-point NRS (95% CI: −0.73 to 0.14). After 2 y, the intervention group reported 1.6 points less leg pain (95% CI: −2.5 to −1.58). The primary outcome measure was the Oswestry Disability Index.	High
Ferreira et al.^22^	People with chronic nerve-related leg pain. Recruited from the community through newspaper and social media advertisements.	60 (54)	Four sessions in 2 wk of manual therapy and exercises aimed at managing neural mechanosensitivity.	Advice to remain active	After 2 wk, the intervention group reported a mean 3.3 points less disability on the ODI (95% CI: 9.6 to −2.9) and 5.3 points on the PSFS (95% CI: 2.2–8.2). After 4 wk, this was 5.0 points on the ODI (11.0 to −1.1) and 4.7 points on the PSFS (1.7–7.8)	After 2 weeks, the intervention group reported a mean −1.1 points less leg pain on a 0–10 NRS scale (95% CI: −2.3 to 0.1). After 4 wk, they reported −2.4 points less pain (95% CI: −3.6 to −1.2).	Low
Kim et al.^43^	People with chronic cervical radicular pain	30 (30)	Eight week, 3 times per week programme of manual cervical traction with neural mobilisation	Manual cervical traction only	After 8 wk, there was a mean 3.27 points difference between the groups on the NDI (*P* = 0.004)	After 8 wk, there was a mean 1 point difference between the groups on a 0–10 numeric rating scale (*P* = 0.006)	High
Hahne et al.^32^	People with chronic lumbar radicular pain from a disk herniation	54 (49)	10 sessions in 10 wk of a multimodal individualised functional restoration programme with a behavioural component	Two 30-min advice sessions	After treatment, patients in the intervention group reported a mean 7.7 points less disability (95% CI: 0.3–15.1) on the ODI compared to the control group. At 26 wk, the difference was 5.7 (95% CI: −1.7 to 13.1) and at 1 y, 8.2 (95% CI: 0.7–15.6) All in favour of the intervention group	After treatment, patients in the intervention group reported a mean 1.1 points less in leg pain on a 0–10 NRS scale (95% CI: −0.3 to 2.4) compared to the control group. At 26 wk, the difference was 1.2 (95% CI: −0.2 to 2.6) and at 1 y, 0.9 (95% CI: −0.5 to 2.3)	Low
Akkan and Gelececk^1^	People with cervical radicular pain	46 (32)	Fifteen sessions in 4 wk of neck stabilisation exercises, generic neck exercises, hot pack, TENS, and ultrasound treatment with training on postural alignment	As in the intervention group but without stabilisation exercises	After 4 wk, there was a 0.39 points mean difference between groups on the NDI in favour of the intervention group. At 12 wk, this was 0.24 points. Between-group difference was *P* > 0.05 (exact value not stated)	After 4 wk, there was no mean difference between the groups on a 0–10 VAS. After 12 wk, the control group reported 0.21 points less pain. Between-group differences *P* > 0.05 (exact value not stated).	High
Calvo-Lobo et al.^12^	People with nerve related arm pain	105 (75)	One intervention group received 5 sessions per wk for 6 wk of median nerve neural mobilisation. Another intervention group received the same frequency of a cervical lateral glide technique.	Oral ibuprofen.	After 6 wk, the oral ibuprofen group reported 14.4 points less disability on the QuickDASH (95% CI: 8.48–20.23) than the group receiving median nerve mobilisations; and 19.2 points less disability (95% CI: 13.79–24.67) than the group receiving cervical lateral glides.	After 6 wk, measured 1 hr after treatment, the oral ibuprofen group reported a mean 1.8 points less pain on a 0–10 NRS (95% CI: 1.12–2.42) than the group receiving median nerve neural mobilisations; and 2.2 points less pain (95% CI: 1.61–2.69) than the group receiving cervical lateral glides.	High
Dedering et al.^15^	People with mostly chronic cervical radicular pain recruited from a neurosurgical department	144 (73)	A 3 mo, 3 times per week programme of progressive neck-specific training with multiple sessions of cognitive behavioral therapy coaching	A general exercise programme with a single session of cognitive behavioural therapy coaching	After 3 mo, the intervention group reported a mean 2 points less disability on the NDI (95% CI: −6 to 10). After 1 y, this difference was 1 point in favour of the control group (95% CI: 1 to −7).	After 3 mo, the intervention group reported a mean 8 points less arm pain on a 0–100 mm VAS (95% CI: −2 to 18). After 1 y, the difference was 2 points in favour of the intervention group (95% CI: −10 to 14).	Some concerns
Rodriguez-Sanz et al.^65^	People with cervical radicular pain	60 (51)	A 6-week programme, 5 d per week of manual therapist-applied median nerve mobilisation.	Waiting list	After treatment, there was a mean 26.97 points difference on the Quick DASH in favour of the intervention group (95% CI: 33.75–20.20)	After treatment, there was a mean 3.70 point difference on a 0–10 NRS in favour of the intervention group (95% CI: 4.29–3.10)	High
França et al.^25^	People with chronic lumbar radicular pain caused by disk herniation	40 (40)	An 8-week, twice-weekly programme of lumbar stabilisation exercises	The same frequency of treatment with TENS	After treatment, there was a mean 8.4 point difference between the groups on the 44-point functional disability version of the ODI (95% CI: 5.44–11.36) in favour of intervention group.	After treatment, there was a mean 3.3 point difference between the groups on a 0–10 VAS (95% CI: 2.12–4.48) in favour of the intervention group.	Some concerns
Plaza-Manzano et al.^64^	People with lumbar radicular pain caused by disk herniation	32 (32)	An 8-wk, 4 times per week programme of neural mobilisation plus motor control exercises	The same frequency of motor control exercises alone	After treatment, the intervention group reported a mean 0.7 points lower score on the RMDQ but between-group analysis was not provided.	After treatment, the intervention group reported a mean 2.6 points of leg pain on a 0–10 NRS (95% CI: 2.2–3.0). The control group reported 3.2 points leg pain (95% CI: 2.8–3.6). Between-group data not provided.	Some concerns
Satpute et al.^69^	People with subacute and chronic lumbar radicular pain, excluding patients with neuropathic pain on S-LANSS	60 (39)	Six sessions over 2 wk of spinal mobilisation with leg movements plus usual care or TENS and active nerve mobilisations.	Usual care of TENS and active nerve mobilisations alone	After treatment, the intervention group reported a mean 3.9 points less pain than the control group on the ODI (95% CI: 5.5–2.2). At 3 mo, the difference was 5 points (95% CI: 6.5–3.4) and at 6 mo, 4.7 points (95% CI: 6.3–3.1).	After treatment, the intervention group reported a mean 2.0 points less pain than the control group on a 0–10 VAS (95% CI: 1.4–2.6). At 3 mo, the difference was 2.8 (95% CI: 2.2–3.4) and at 6 mo, 2.6 (95% CI: 1.9–3.2).	Some concerns
Basson et al.^7^	People with acute and subacute nerve-related arm pain, without “serious neurological signs.”	86 (78)	Usual care of spinal mobilisations, exercise, and advice to stay active plus therapist-applied neural mobilisation.	Usual care of neck mobilisations, exercise, and advice to stay active.	After 6 wk, the intervention group reported a mean 0.5 points more disability on the PSFS (95% CI: −3.9 to 3.1). After 6 mo, the difference was −0.1 (95% CI: −2.7 to 2.5) and at 12 mo, −0.3 (95% CI: −2.7 to 2.5).	After 6 wk, the intervention group reported a mean 0.6 points less pain on a 0–10 point NRS (95% CI: −0.6 to 1.8). After 6 mo, the difference was 1.1 (95% CI: 0.1–2.2) and at 12 mo, 1.1 (0.1–2.0).	High

Unless otherwise specified, studies used pain as a primary outcome measure. The number of participants shown at follow-up is for the longest-term follow-up in each trial.

95% CI, 95% confidence interval; GP, general practitioner; NDI, Neck Disability Index; NRS, Numeric Rating Scale; ODI, Oswestry Disability Index; PSFS, Patient-Specific Functional Scale; QuickDASH, Quick disabilities of the Arm, Shoulder and Hand Score; RMDQ, Roland Morris Disability Questionnaire; S-LANSS, Self-Administered Leeds Assessment of Neuropathic Symptoms and Signs; VAS, visual analogue scale; TENS, transcutaneous electrical nerve stimulation.

### 3.1. Stabilisation and motor control interventions

**Figure 1.**
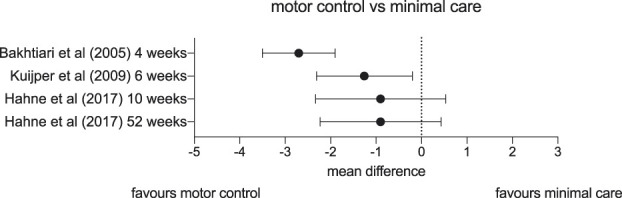
Forest plot depicting mean differences in pain (on visual analogue scales or numerical pain rating scales) and confidence intervals of studies comparing motor control and stabilisation exercises vs minimal care.

Most trials that examined the effect of interventions to stabilise or improve the control of the spine against no or minimal care found evidence of some benefit (Fig. 1). First, Bakhtiary et al.^4^ (n = 60) tested stabilisation exercises in 60 patients with lumbar radicular pain who had been referred to an orthopaedic department. One group began their exercise programme immediately after enrolment in the trial and continued for 4 weeks. The other group waited for 4 weeks after their enrolment and then performed the same exercise programme. After the first 4 weeks, the group who had been exercising reported a 2.7-cm reduction of pain on a 10-cm visual analogue scale (95% confidence interval [CI]: −3.5 to −1.9) compared to the control group, a clinically relevant difference. At the end of the trial, when the second group had exercised, pain scores converged. Second, Kuijper et al.^48^ ran a 3-arm trial with participants with acute cervical radicular pain (n = 205). They tested the effects of twice-weekly supervised stabilisation and mobilisation exercises with a home exercise programme, a semihard neck collar, and minimal care. Arm pain improved in all groups, but both the exercise and neck collar groups outperformed the minimal care group at 6 weeks by a clinically meaningful difference. By 6 months, there was no difference between the 3 groups. Although there was no evidence of difference at any time point between the exercise and the neck collar groups, Kuijper et al. recommended the neck collar over exercise based on time- and cost-efficiency. Third, Hahne et al.^32^ (n = 54) published a prespecified subgroup analysis of a larger trial that compared extensive care to minimal care for people with lumbar radicular pain. Patients received either 10 sessions over 10 weeks of individualised functional restoration (including motor control but also functional exercises cognitive behavioural therapy, and pacing advice) or two 30-minute advice sessions. There was no clinically meaningful mean difference in leg pain between the 2 groups at any time point up to and including one-year follow-up. However, there is some uncertainty in these results because the confidence intervals include a wide range of plausible differences. Nevertheless, considering this trial compared an extensive intervention to minimal care, it is unlikely these results represent a clinically worthwhile effect.

**Figure 2.**
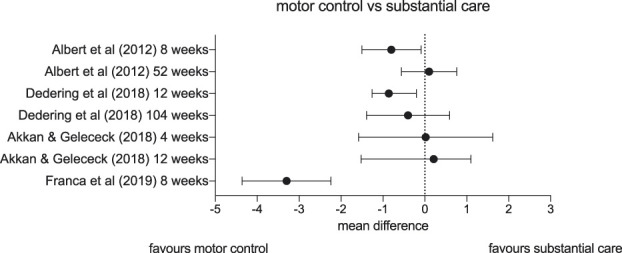
Forest plot depicting mean differences and confidence intervals in pain (on visual analogue scales or numerical pain rating scales) of studies comparing motor control and stabilisation exercises vs substantial care.

Most trials that compare stabilisation or motor control exercises to more substantial treatment do not report any clinically meaningful superiority for these exercises (Fig. 2). Albert and Manniche^2^ (n = 181) tested a combination of stabilization exercises and McKenzie exercises (which are based on the participant's preferred direction of movement) against a general exercise programme designed to increase heart rate for patients with lumbar radicular pain. Both groups also received education and advice. At short-term follow-up, there was a small, not clinically meaningful difference between groups in favour of the intervention arm, but this was not observed at long-term follow-up. In a similar trial with similar results, Dedering et al.^15^ (n = 144) compared a progressive neck-specific exercise programme to a general exercise programme, both provided alongside cognitive behavioural therapy-informed coaching for patients with cervical radiculopathy. This study also did not observe any clinically meaningful difference between groups at any time. Akkan and Gelececk^1^ (n = 46) examined the effect of adding stabilisation exercises to a programme of electrotherapy, stretching, and isometric exercises. Both groups improved, with no clinically meaningful difference in pain scores at any time point between groups. Finally, in the only study in this category to find meaningful benefit for motor control exercises over a comparator, França et al.^25^ (n = 40) tested sixteen 60-minute sessions of motor control exercises against the same duration of transcutaneous electrical nerve stimulation (TENS) over an 8-week period, for patients with lumbar radicular pain. Both groups improved, but patients in the motor control group reported 3.3 points less pain on a 10-point scale than the TENS group (95% CI: −2.12 to −4.48).

To summarise, 3 trials investigating stabilisation or motor control exercises reported a clinically meaningful difference in pain—2 compared to no treatment^4,48^ and one compared to TENS.^25^ Five trials found no evidence of a clinically meaningful benefit compared to comparators of a neck collar,^48^ advice,^32^ general exercise,^2,15^ or when added to usual care.^1^ This suggests that exercises directed at improving the stability or control of spinal movement might provide clinically meaningful benefit over minimal care. However, the only study in this group with low risk of bias, Hahne et al., contradicts this.^32^ There is no evidence that these kinds of exercises are superior to more substantial interventions, including general exercise, for people with radicular pain. This is consistent with the literature for low back pain.^72^

### 3.2. Interventions directed at neural mechanosensitivity

Interventions directed at neural mechanosensitivity were the most common type of intervention studied for radicular pain. Commonly used physiotherapeutic techniques addressing neural mechanosensitivity include specific movements of peripheral nerves in relation to their surrounding tissues (eg, neural sliders or tensioners) or interface techniques that are directed at the tissue surrounding the nerve.^11^ These can be performed as exercises by the patient or as a form of manual therapy by the practitioner.

**Figure 3.**
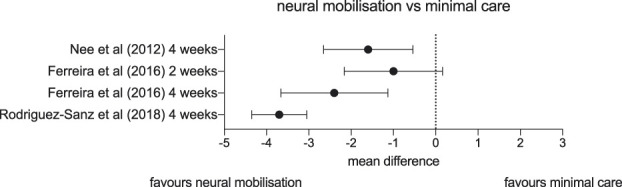
Forest plot depicting mean differences and confidence intervals in pain (on visual analogue scales or numerical pain rating scales) of studies comparing neural mobilisation interventions vs minimal care.

The trials that compared neural tissue management to no care or minimal care all found beneficial effects of neural tissue management (Fig. 3). First, Nee et al.^61^ (n = 60) tested neural tissue-based manual therapy, exercise, and education against advice to continue usual activities for people with nerve-related neck/arm pain. The trial specifically excluded patients with more than 2 abnormal findings on their neurological examination, making it unlikely that patients with radiculopathy were included. After 4 sessions in 4 weeks, there was a clinically meaningful 1.5-point difference in arm pain on an 11-point numeric rating scale (NRS) favouring the intervention group (95% CI: −2.6 to −0.5). Second, Ferreira et al. published a similar trial^22^ (n = 60) comparing 4 sessions of neural tissue management in 2 weeks to advice. At their primary endpoint of 2 weeks, there was a 1.1-point difference in pain on an 11-point NRS between the 2 groups with a confidence interval including no effect (95% CI: −2.3 to 0.1). At 4 weeks, there was a difference of 2.4 points in favour of the intervention group with a 95% confidence interval indicating data consistent with a true difference in means between −3.6 and −1.2 points. Third, a multicentre RCT by Rodriguez-Sanz et al.^65^ (n = 60) compared an intensive course of median nerve mobilisation to no treatment in participants with cervical radicular pain. Patients in the intervention group reported a 3.7-point decrease in pain (95% CI: −4.29 to −3.10) from baseline on an 11-point NRS after 6 weeks of treatment compared to the control group. This is a large between-group difference for a rehabilitation trial. This may be because in this study, there was a particularly large difference between groups in therapist contact time. The intervention group had 5 physiotherapy sessions per week for 6 weeks, whereas the control group had no contact with a physiotherapist. Of note, this trial was found to have high risk of bias.

**Figure 4.**
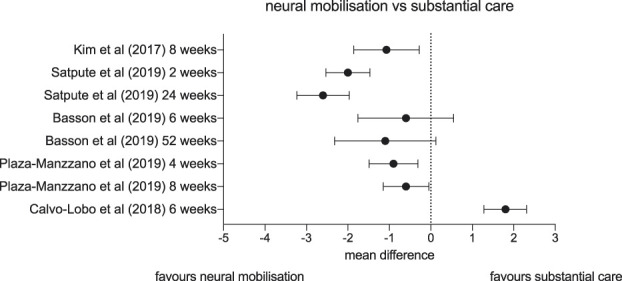
Forest plot depicting mean differences and confidence intervals in pain (on visual analogue scales or numerical pain rating scales) of studies comparing neural mobilisation interventions vs substantial care.

Trials that examine the efficacy of adding neural tissue management to more substantial care reported mixed results (Fig. 4). Two of these found some benefit of neural tissue management in reducing pain. Kim et al.^43^ (n = 30) compared neural mobilisation with manual cervical traction to mechanical cervical traction alone for patients with cervical radicular pain. After 8 weeks of treatment, 3 times per week, the intervention group reported one-point lower pain score on an 11-point NRS. Satpute et al.^69^ (n = 60) examined the effects of adding leg mobilisations with movement to usual care of neural tissue management, exercise, and TENS for patients with subacute and chronic lumbar radicular pain. This intervention is based on the Mulligan approach of manual therapy. After 6 sessions over 2 weeks, the intervention group reported 2.0 points less pain on a 10-cm visual analogue scale compared to the usual care group (95% CI: −1.4 to −2.6), and this difference was sustained at 6-month follow-up (mean difference −2.6; 95% CI: −1.9 to −3.2). This was the only trial without high risk of bias to demonstrate clinically meaningful change at long term follow-up. However, the results reported by Satpute et al. might not generalise to all patients with radicular pain because the authors specifically screened out patients with neuropathic pain, as measured by LANSS, and patients who did not experience an initial positive response to the intervention.

Three trials that added neural tissue management to more substantial care did not find clinically meaningful benefits of neural tissue management. First, Basson et al.^3^ (n = 86) was the only study to examine neural massage, which the authors added to spinal mobilisations, exercise, and advice for patients with recent-onset cervical radicular pain. At 6 weeks, there was a mean 0.6-point difference in pain on an 11-point NRS between the 2 groups with a confidence interval including no effect (95% CI: 0.6 to −1.8). At 6 months, the difference between the groups was a clinically significant −1.1 points, with a wide range of plausible true differences (95% CI: −0.1 to −2.2). Second, a small trial by Plaza-Manzanno et al.^64^ (n = 32) did not find evidence for a clinically meaningful difference in pain between a group of patients with lumbar disk herniation who received 8 sessions of manual neurodynamic mobilisation plus motor control exercises, compared to a group who received motor control exercises alone. Finally, a three-arm trial by Calvo-Lobo et al.^12^ (n = 105) found that 2 types of manual neural mobilization were inferior to oral ibuprofen in patients with cervical radicular pain.

In summary, neural tissue management seems to reduce pain compared to no or minimal treatment. However, when compared to more substantial treatment, the results were more mixed and there is not enough evidence to make strong conclusions. Our equivocal conclusions are echoed in 2 recent systematic reviews. Su and Lim^74^ reported that neural tissue management is more effective than minimal care for nerve-related musculoskeletal pain, but has not been demonstrated to be superior to other interventions. Basson et al.^7^ grouped conditions by aetiology and found that neural tissue management is effective for nerve-related low back pain and nerve-related neck and arm pain, but did not comment on whether it is superior to other interventions. Both systematic reviews included RCTs we did not identify in our literature search because we limited our search to PubMed and restricted years of publication.

### 3.3. Other interventions

The remaining RCTs that examined the efficacy of physiotherapy on reducing radicular pain investigated a heterogeneous group of interventions.

**Figure 5.**
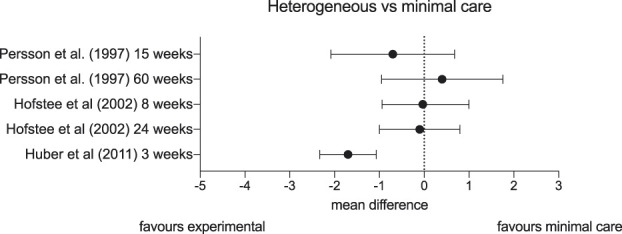
Forest plot depicting mean differences and confidence intervals in pain (on visual analogue scales or numerical pain rating scales) of studies comparing a range of heterogeneous interventions vs minimal care.

Several trials tested physiotherapy interventions against minimal care with mixed results (Fig. 5). Persson et al.^63^ (n = 82) compared physiotherapy consisting of TENS, hot and cold packs, massage, and active aerobic exercise, to a hard cervical collar for patients with cervical radicular pain. Unlike the similar trial by Kuijper et al.,^48^ Persson et al. recruited patients with chronic rather than acute pain. At 4 months, physiotherapy did not confer a clinically meaningful benefit over a cervical collar. Hofstee et al.^35^ (n = 250) used a 3-arm trial design and also did not find differences between physiotherapy (including manual mobilisations and spinal exercises), bed rest, and advice to continue with activities of daily living for people with acute lumbar radicular pain. Finally, a trial by Huber et al.^36^ (n = 52) found a small benefit from early isometric exercise compared to advice alone for acute lumbar radicular pain (n = 52).

**Figure 6.**
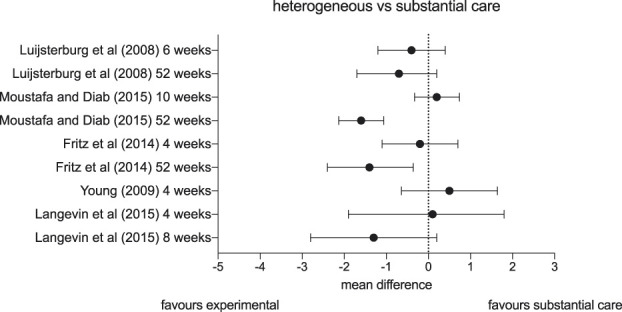
Forest plot depicting mean differences and confidence intervals in pain (on visual analogue scales or numerical pain rating scales) of studies comparing a range of heterogeneous interventions vs substantial care.

There were also mixed results from trials examining the effect of adding heterogeneous physiotherapy interventions to more substantial care (Fig. 6). Luijsterburg et al.^53^ conducted a trial examining the effect of exercise in patients with lumbar radicular pain. The authors randomised 135 people with acute and subacute lumbar radicular pain into 2 groups. One group remained in general practitioner care and the other group additionally saw physiotherapists who prescribed exercises aimed at return to activity. There was no clinically meaningful difference in pain between the groups at any time point up to one year, with confidence intervals consistent with no true difference between the 2 groups. Moustafa and Diab^59^ published a similarly pragmatic trial with long-term follow-up that aimed to examine the effect of forward head posture corrective exercises on lumbar radicular pain (n = 154). Both groups received 10 weeks of progressive and relatively intensive functional restoration training with cognitive behavioural therapy-informed coaching. In addition, the intervention group was shown exercises to correct forward head posture. At the end of the intervention, there was no clinically meaningful difference between groups. But, after 2 years, the group who underwent neck posture training reported 1.6 points less leg pain on an 11-point NRS. The data suggest that the control group “relapsed,” whereas the intervention group maintained their improvement. These results are clinically meaningful but should be treated with caution, given the study's high risk of bias, the low plausibility of neck posture training for lumbar radicular pain, and the unusual pattern of treatment effectiveness manifesting only 2 years after an intervention. Two trials examined traction for cervical radicular pain. Fritz et al.^26^ (n = 86) found that adding mechanical or manual traction to a shoulder and neck strengthening exercise programme compared to the same strengthening programme alone resulted in clinically meaningful pain reduction for patients with cervical radicular pain in the long-term follow-up, but not immediately after treatment. In a similar trial, Young et al.^79^ (n = 81) added traction to manual therapy and exercise for patients with the same condition and reported no clinically meaningful difference in arm pain in the short term. Finally, Langevin et al.^49^ (n = 36) tested whether manual therapy directed at increasing space in the intervertebral foramen to reduce pressure on a nerve root is more effective than manual therapy without this intention in patients with cervical radicular pain. After 8 sessions in 4 weeks, including a home exercise programme in both groups, there was no clinically meaningful difference on a 10-point NRS between the groups (mean difference 0.1; 95% CI: −1.9 to 1.8) and after 8 weeks, the difference between the groups was −1.3 points; however, the range of treatment effects included no difference (95% CI: −2.8 to 0.2).

### 3.4. Effects of physiotherapy on disability for radicular pain

Although patterns for the effectiveness of physiotherapy on pain and disability were similar, disability seemed to improve to a lesser extent, and less often, than pain (for full disability-related results, please see Table 2). For example, Nee et al.,^61^ Kim et al.,^43^ and Satpute et al.^69^ reported clinically meaningful changes in pain but not disability.^43,61,69^ Ferreira et al.^22^ did find a meaningful change in the Patient-Specific Functional Scale, but not in the Oswestry Disability Index. No studies reported a meaningful change in disability without a comparable effect for pain. This mirrors the findings of a recent review that found that for people with chronic pain, disability might be more resistant to change than pain.^68^

### 3.5. Safety of exercise for radicular pain

Adverse event reporting was inconsistent. Eleven of 23 trials did not record adverse events. Ten trials did record adverse events but without describing how these were defined or how information about them was collected. Just 2 trials provided a detailed description of how adverse events were defined and recorded. Both suggested that mild adverse events were quite common. Nee et al.^61^ reported that 42% of patients experienced an adverse event after treatment targeted at reducing neural mechanosensitivity, mostly aggravation of neck or arm pain, 95% of which subsided within 24 hours. Fritz et al.^26^ reported that 56% of participants experienced adverse events, mostly worsening neck pain, with no difference in adverse event rate between the 3 trial arms. No serious or lasting adverse events were recorded in any study.

### 3.6. Summary and future direction for physiotherapy in radicular pain

To summarise, trials investigating the effects of physiotherapeutic treatments for radicular pain mostly investigate stabilisation and motor control exercise or neural tissue management, with only occasional studies using other techniques. Four trials had a low risk of bias.^22,32,53,61^ Two of these, the studies by Nee et al.^61^ and Ferreira et al.,^22^ suggested that neural mobilisations improved pain more than minimal care, but reported confidence intervals that included a wide range of plausible effects. Two did not find evidence of a clinically meaningful effect: Hahne et al.,^32^ when comparing an individualised functional restoration programme including stabilisation exercises to minimal care, and Luijsterburg et al.,^53^ who compared general functional exercises to general practitioner care alone. Taken together, we conclude that interventions aimed at reducing neural mechanosensitivity may be more promising in reducing radicular pain than those aimed at spinal control and stability. However, this notion is based on their comparison to other interventions, with no direct comparison between these interventions. Also, the clinical importance of this benefit remains unclear. Conflicting results from mixed quality trials make it impossible to draw strong conclusions.

Our equivocal conclusions are consistent with the most relevant recent systematic reviews. In addition to those mentioned above,^7,74^ Fernandez et al.^21^ found low-quality evidence that exercise provides a small benefit for pain over advice to stay active in the short term and moderate-quality evidence of no difference in the medium term in patients with lumbar radicular pain. Liang et al.^50^ found that exercise is more effective than sham for radicular pain, but that this was only observed in Chinese studies, whereas Western studies reported no benefit.

## 4. Implications for practice, remaining challenges, and potential ways forward

Although there is some evidence that physiotherapy treatments provide meaningful improvements in pain, QoL, and disability for people with peripheral neuropathies and neuropathic pain, there are also high-quality studies in which this does not happen. In fact, some of the available studies found that physiotherapy interventions did not improve pain to a greater extent than more simple and less time-intensive treatments such as ibuprofen,^12^ a neck collar,^48^ or general practitioner care alone.^53^ Where improvements do occur, they are often modest.

On the positive side, physiotherapeutic treatments do seem to be safe for people with CIPN and radicular pain. This means that people with these neuropathies can take part in exercise and enjoy its social, recreational, and health benefits. This is particularly important for people with cancer for whom exercise is strongly recommended because it has well-proven benefits beyond pain reduction.^57^ Nevertheless, when it comes to the benefits of exercise for peripheral neuropathic pain itself, we would advise physiotherapists not to set patients' expectations, or their own, too high.

The results of our review also invite discussion on what should be the priority in a physiotherapy appointment. Although we have concentrated on the effects of exercise and manual therapy, usually studied as distinct interventions, physiotherapy often assumes a broader role when treating patients with peripheral neuropathic pain and neuropathies. Physiotherapists assess and monitor patients' neuropathies and their wider health, educate them about their condition, coach them through return to work and valued activities, and help them make sense of their predicament. Given the modest effect of the exercise and manual therapy interventions on peripheral nerve pain and disability studied to date, physiotherapists should consider whether it is most valuable to use their time with a patient for these interventions or for those other aspects of their role. That said, there is an absence of evidence that those other aspects of a physiotherapist's role confer a meaningful benefit to patients with peripheral nerve pain. Therefore, we suggest this as a direction for further studies.

### 4.1. Quality and design of studies

The quality of available studies is mixed, with only 4 of 31 studies reported here having low risk of bias.^22,32,53,61^ The greatest source of risk of bias was possible selective outcome reporting because some studies did not have registered protocols and of those that did, many were retrospectively registered or lacked detail. The second greatest source of risk of bias was concerns about the randomisation process. There were also examples of overinterpretation of results in the presence of methodological shortcomings. Although most studies reported adverse events, only 4 provided details on how adverse events were defined and measured. Better descriptions of the interventions and their dosage are also required. For instance, few studies documented whether exercise and manual therapy was performed in a pain-free range or “into pain.” With improving quality of future studies, more conclusive recommendations on the efficacy and safety of physiotherapy for peripheral neuropathic pain can be made.

Although we have focused on RCTs in this review, it may be prudent to consider alternative approaches to evaluate potential benefits of physiotherapy for patients with peripheral neuropathies. For instance, some large prospective cohort studies have been useful in understanding the relationship between exercise and symptoms of CIPN.^30,58^ Longitudinal observation of large patient cohorts would provide important information in addition to clinical trials.

### 4.2. Could we learn from preclinical science?

In some trials, exercise and manual therapy do not help peripheral nerve pain, and if they do help, benefits are often small. In this sense, physiotherapeutic treatments are similar to pharmacotherapeutic ones, for which effects are also modest.^23^ The absent or small effect sizes suggest that the physiotherapy interventions as currently packaged are not particularly effective. Most physiotherapeutic interventions are based on current clinical practice, which largely follows empirical concepts. We propose that the field may benefit from promising results in preclinical sciences. For instance, there is a strong body of preclinical literature suggesting that exercise is not only hypoalgesic, but also “neuroprotective,” or “neuroregenerative,” after focal and systemic nerve injury. Exercise as a means of neuroprotection or neuroregeneration is an interesting paradigm that could be explored in future clinical research.

The 2 main types of exercises examined in the preclinical literature are aerobic (eg, running and swimming) and passive neurodynamic treatments. There is convincing evidence that aerobic exercise not only reverses established neuropathic pain,^31^ but also prevents its development.^29^ Aerobic exercise seems more beneficial than resistance exercise for motor nerve regeneration and functional recovery, although this remains to be confirmed for sensory measures.^35^ The beneficial effects of aerobic exercises have been attributed to a regulation of inflammatory cytokines, neurotrophins, neurotransmitters, endogenous opioids, ion channel function, and descending inhibition as well as axonal and myelin regeneration capacity.^31,40^ Intriguingly, the neuroprotective benefits of aerobic exercise are influenced less by the type of exercise (eg, swimming vs running) than by other variables. For example, exercise dosage affects neuroprotection: it seems that low-to-moderate intensity exercise is neuroprotective but moderate-to-high intensity exercise can be neurotoxic.^13,14^ Timing seems also to be important. In rodents, exercising as soon as a week after nerve injury is safe and seems more beneficial than delayed onset of exercise.^52^

Similar to aerobic exercise, neurodynamic exercises and joint mobilisations seem to induce hypoalgesia and improve nerve regeneration in preclinical models of neuropathic pain. Although most studies have to date been performed in focal nerve injury (eg, sciatic nerve injury),^28,54,66,67,71^ there is now evidence that neurodynamic exercises may also attenuate mechanical allodynia in preclinical models of diabetic neuropathy.^80^ Similar to aerobic exercises, the passive neurodynamic treatments seem to exert their benefit through powerful effects on the inflammatory response, nerve regeneration, and remyelination as well as the opioid system. Intriguingly, the preclinical studies used end-of-range passive neural tensioner exercises, which are arguably more aggressive than the slider exercises used in most human studies.

These preclinical findings highlight some priority areas that may need reconsideration in clinical practice. First, we may need to rethink the time of onset, dosage, and intensity of aerobic exercise to optimise the promising albeit small effects in patients with systemic peripheral neuropathies. Second, given the potent neuroprotective and hypoalgesic preclinical effects of aerobic exercise, its efficacy in the management of neuropathic pain needs to be evaluated. Although this has started to happen for CIPN, data are still missing for radicular pain. Such studies might take a simple kind of tolerable aerobic exercise and examine the effects of dosage, timing, and adherence on outcomes. This would be a break from “traditional physiotherapy” for radicular pain intervention research, but fully consistent with preclinical science. One potential limitation of such an approach is the tolerability of exercise especially in patients with acute radicular pain. It remains to be shown whether the robust preclinical evidence for neuroprotective and hypoalgesic effects of aerobic exercise translates to patients. Unfortunately, such translation has often failed in the pharmacological literature.^77^ Third, we have to explore the discrepancy of more aggressive mobilisations used in preclinical models vs more gentle exercises in patients. Such studies may first be evaluated for safety in experimental models of neuropathic pain before they are translated to patients. Importantly, if more aggressive clinical interventions are trialed, careful recording and reporting of adverse events is required.

### 4.3. Patient selection

Another explanation for the small effect sizes of physiotherapeutic interventions for patients with neuropathic pain might be the heterogeneous pathomechanisms that contribute to nerve pain and which may require distinct treatment approaches. For example, CIPN might arise from microtubule disruption, demyelination, and preferential small fiber neuropathy among other mechanisms. Similarly, radicular pain might arise from a combination of nerve root compression, neuroinflammation, ischaemia, central sensitization, and other mechanisms. In addition, not all patients with spinally referred pain have frank neuropathic pain^47,55^ with patients presenting somewhere on a continuum between “purely nociceptive” and “purely neuropathic” pain.^70^

In response to this heterogeneity, an approach called sensory phenotyping has been proposed.^5^ This approach uses a detailed sensory examination and questionnaires to identify common patterns of signs and symptoms that serve as surrogate markers for distinct pathomechanisms. For example, a recent article identified 3 consistent phenotypes across multiple neuropathic pain conditions, including radicular pain: sensory loss, thermal hyperalgesia, and mechanical hyperalgesia phenotypes.^6^ These phenotypes are suggested to reflect deafferentation, peripheral sensitisation, and central sensitisation, respectively. Stratification according to sensory phenotype has shown promising results in the pharmacological treatment of patients with neuropathic pain of different aetiologies, although its benefits have not yet been consistently demonstrated.^24^

We did not find any physiotherapy studies that engaged with the phenotypes identified by large cohort studies. Also, most studies were not powered to perform post hoc analyses to identify responding phenotypes. A reasonable hypothesis would be that radicular pain characterised by the “sensory loss” phenotype might respond better to aerobic exercise, which effectively stimulates axonal regeneration in preclinical studies.^14,17^ By contrast, the “mechanical hyperalgesia” phenotype has been associated with the presence of central mechanisms, thus potentially benefiting from management targeted at these. An important limitation of a phenotyping approach is that it requires large sample sizes, which seems to be a main challenge in the existing literature.

### 4.4. Limitations

This review aimed to provide the reader with a broad overview of the available literature on physiotherapy for people with peripheral neuropathies. We therefore chose a narrative design. This approach may have missed some relevant studies, particularly because we searched in one database only and limited our search to studies written in English. Furthermore, because this is a narrative review, each stage of the process of searching the literature, selecting the included studies, extracting data, and assessing risk of bias was performed by a sole investigator.

Because of the scope of this review, we focused on exercise and manual therapy, which are recommended treatment options in the current guidelines on management of patients with lumbar radicular pain^62^ and included in most undergraduate curricula. Finally, we would like to emphasise that we were concerned with effects on pain and disability/QoL as important outcome measures in patients with neuropathic pain. Future work is required to examine whether alternative physiotherapy interventions may be beneficial in this patient population and whether physiotherapy may beneficially influence other relevant outcomes such as sleep or emotional wellbeing.

Despite the limitations of this review, we hope we have succeeded in providing the reader with a broad overview of the relatively novel field of physiotherapy for peripheral neuropathic pain.

## 5. Conclusions

Currently available data suggest that physiotherapy may reduce symptoms and improve QoL in patients with already established CIPN. However, there is a lack of evidence for its preventative effect in patients who do not yet have symptoms. For radicular pain, treatments aimed at improving motor control and reducing neural mechanosensitivity seem to be better in reducing pain compared to no or minimal treatment. However, results were equivocal in studies comparing these interventions to more substantial treatments. In radicular pain, disability seemed to improve to a lesser extent, and less often, than pain. Of note, adverse events from physiotherapy seemed rare; however, these were not consistently reported across all studies.

Although it is encouraging to see that the scientific exploration of the efficacy of physiotherapy for patients with neuropathic pain is growing, the available evidence is limited and of mixed quality. We therefore recommend caution regarding any firm treatment recommendations. The in-part promising preliminary data warrant further exploration of physiotherapy in the treatment of patients with peripheral neuropathies. These efforts may be guided by preclinical work and benefit from more detailed patient phenotyping.

## Disclosures

The authors have no conflict of interest to declare.

## Appendix A. Supplemental digital content

Supplemental digital content associated with this article can be found online at http://links.lww.com/PR9/A70, http://links.lww.com/PR9/A68 and http://links.lww.com/PR9/A69.
